# Angiomatoid fibrosis histiocytoma in the pulmonary artery: A case report

**DOI:** 10.1111/1759-7714.13929

**Published:** 2021-03-13

**Authors:** Shuji Mishima, Osamu Mishima, Koji Azuhata, Hisashi Shimojo, Nobuo Ito, Kenji Misawa, Kimihiro Shimizu

**Affiliations:** ^1^ Department of General Surgery Aizawa Hospital Matsumoto Japan; ^2^ Department of Anatomic Pathology Aizawa Hospital Matsumoto Japan; ^3^ Division of General Thoracic Surgery, Department of Surgery Shinshu University School of Medicine Matsumoto Japan

**Keywords:** angiomatoid fibrosis histiocytoma, case report, EWSR1‐CREB1 fusion, pulmonary artery

## Abstract

Angiomatoid fibrosis histiocytoma (AFH) is a rare neoplastic disease. Only one report has demonstrated an intraluminal tumor of the pulmonary artery (PA) corresponding to AFH to date. We describe the case of AFH with *EWSR1‐CREB1* fusion occurring in the ascending artery. A 42‐year‐old man exhibited an abnormal nodule on chest computed tomography (CT) during checkup. It revealed an intraluminal mass in the ascending artery with significant metabolic uptake in positron emission tomography (PET)/CT. Therefore, right upper lobectomy with wedge resection of the PA trunk was performed. Histologically, the tumor was multinodular and surrounded by a dense lymphoplasmacytic cuff. Each nodule was composed of myxoid stroma and comprised ovoid or spindle cell fascicles with mild atypia. Fluorescent in situ hybridization (FISH) analysis confirmed *EWSR1‐CREB1* fusion. A diagnosed as AFH was made. This report widens the spectrum of differential diagnoses of primary tumors occurring in the PA.

## Graphical abstract

Angiomatoid fibrosis histiocytoma (AFH) is a rare neoplastic disease. Only one report has demonstrated an intraluminal tumor of the pulmonary artery trunk corresponding to AFH with ESWR1‐ATF1 fusion to date. This is the first reported case of AFH occurring in the ascending artery with EWSR1‐CREB1 fusion.

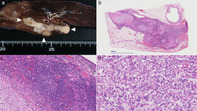

## INTRODUCTION

AFH is a rare neoplastic disease that usually occurs in the deep dermis or subcutis of the extremities in children and young adults. It was first reported as an angiomatoid malignant fibrous histiocytoma by Enzinger in 1979.^1^ It rarely occurs in extrasomatic sites,^2^ and only one report has revealed an intraluminal tumor of the PA trunk corresponding to AFH with *EWSR1‐ATF1* fusion.^3^ Herein, we describe a first case of AFH with *EWSR1‐CREB1* fusion occurring in the ascending artery.

### Case report

**FIGURE 1:**
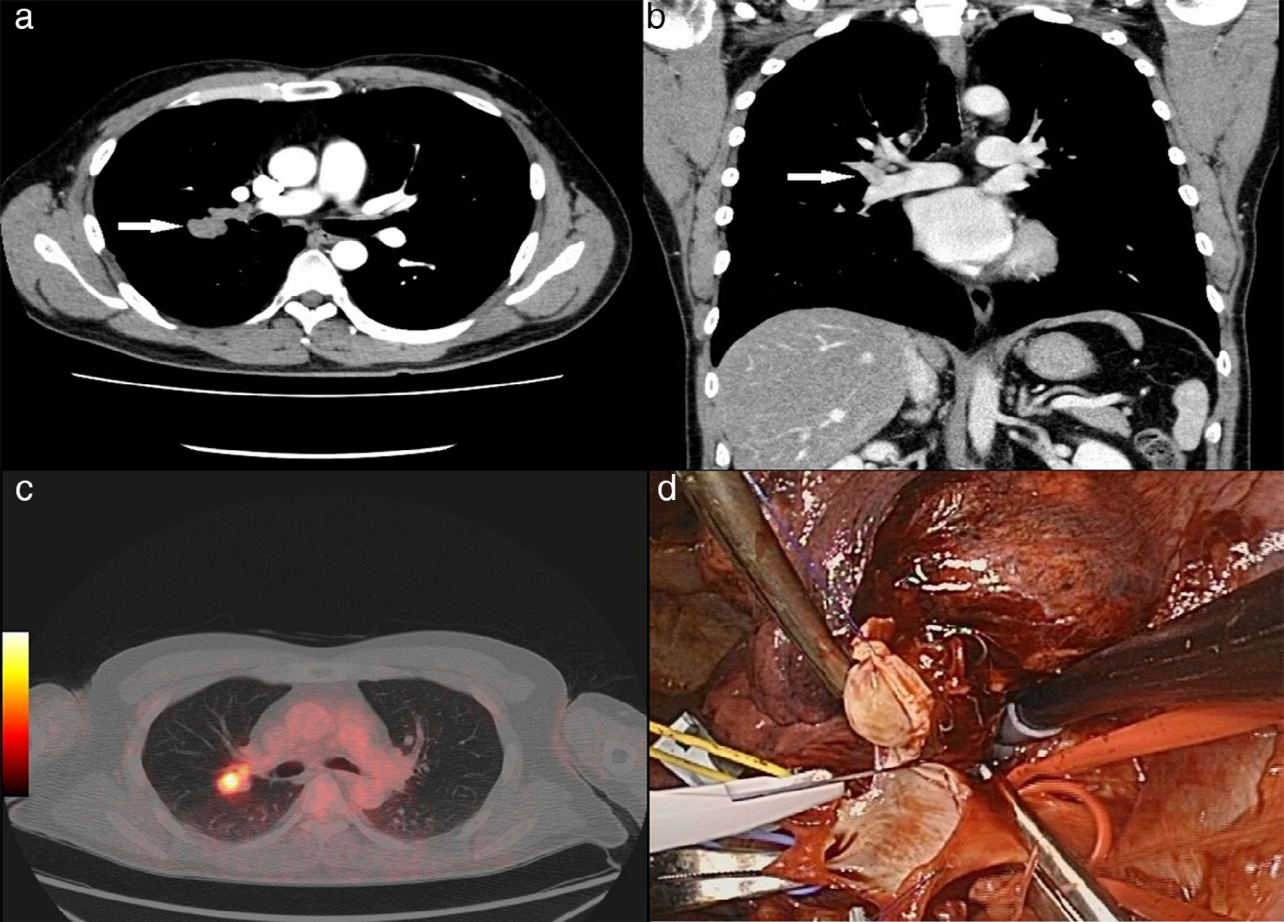
(a), (b) Enhanced chest CT showed an intraluminal mass in the brunch of right pulmonary artery (arrowhead). (c) PET/CT scan revealed significant ^18^FDG uptake, the FEVmax was 7.8. (d) Right upper lobectomy with angioplasty was undertaken

The patient was a 42‐year‐old male who was referred to our institution because of an abnormal nodular shadow on chest computed tomography (CT) during checkup. Enhanced chest CT showed an intraluminal mass in the ascending artery (Figure 1(a),(b)). This lesion was well delineated, solid, and moderately hypervascularized. Positron emission tomography (PET)/CT revealed significant ^18^FDG uptake (Figure 1(c)). Clinical examination and biochemical blood analyses did not reveal any abnormalities. Endobronchial ultrasound‐guided fine needle aspiration was performed, but no specimen was obtained. Due to the significant ^18^FDG uptake, some aggressive malignancies were suspected. At a multidisciplinary consensus meeting, a decision was made to perform right upper lobectomy with angioplasty (Figure 1(d)). No recurrence was detected until the 6 months postoperative follow‐up.

**FIGURE 2:**
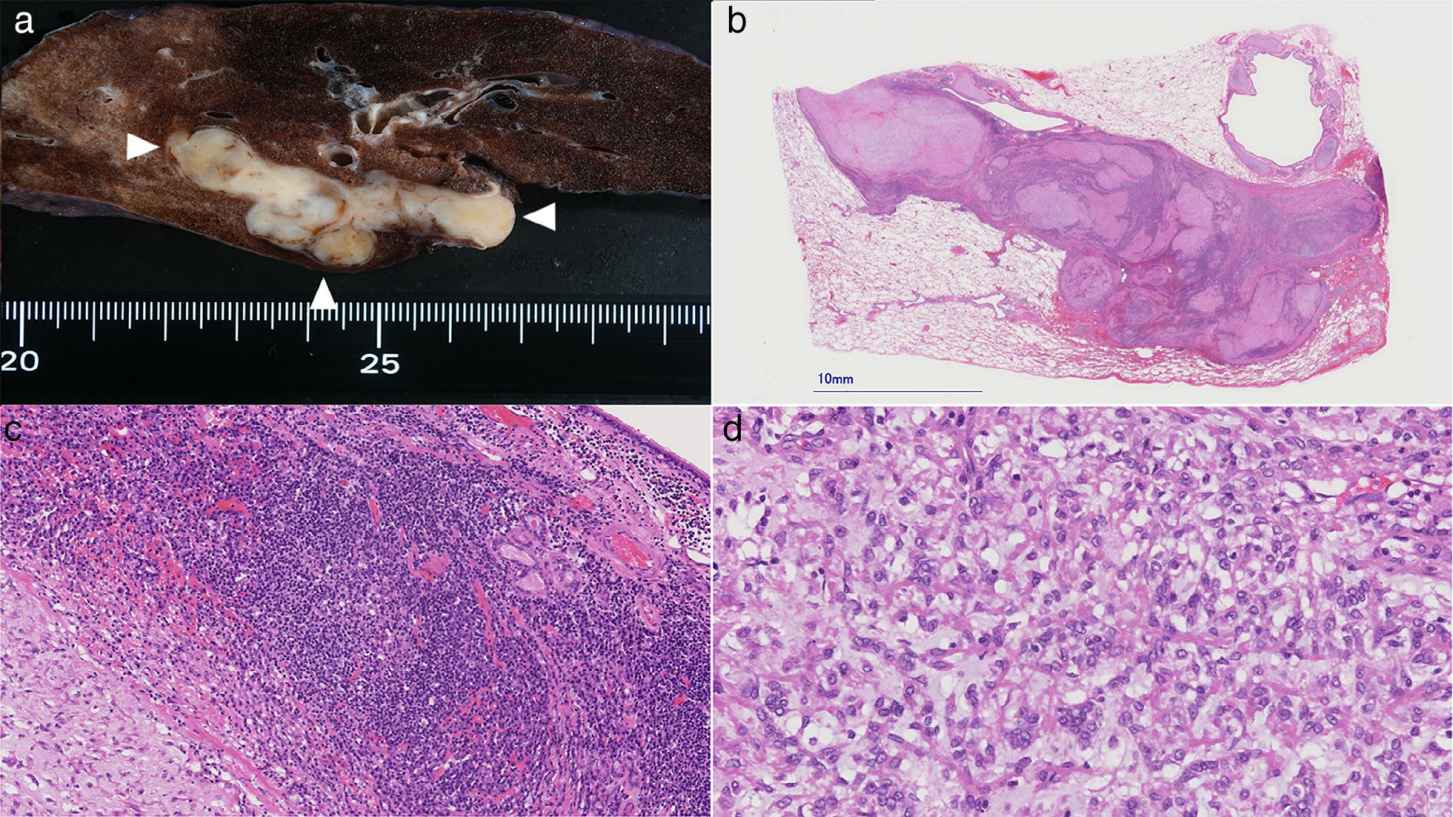
(a) The mass appeared white and solid, measuring 50 × 18 × 15 mm in the ascending artery, involving the whole vascular wall (arrow heads). (b) The tumor was multinodular and (c) surrounded by a dense lymphoplasmacytic cuff. (d) Each nodule comprised fascicles of ovoid or spindle cells with mild atypia

**FIGURE 3:**
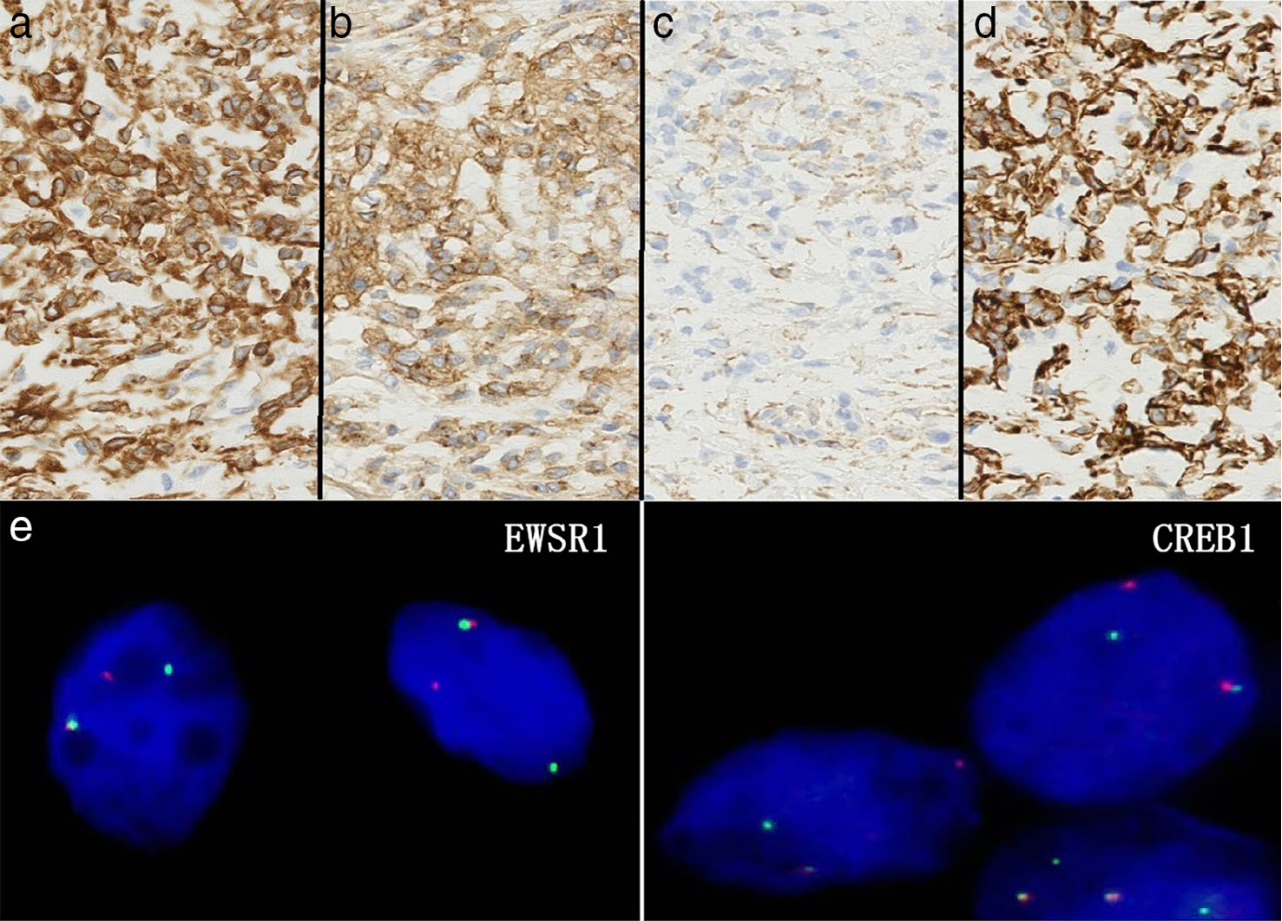
Immunohistochemically, spindle cells were positive for (a) vimentin, (b) CD99, (c) CD68, and partially positive for (d) desmin. (e) EWSR1, CRBE1, and ATF1 gene FISH analysis was performed and exhibited split signals for 70%, 82%, and 0% respectively

A right upper lobe with a part of the right PA was resected. The ascending artery was narrowed by a mass measuring 50 × 18 × 15 mm, involving the whole vascular wall (Figure 2(a)). In the cross‐section (Figure 2(b)–(d)), the mass appeared white and solid. On histological examination, the tumor was multinodular and surrounded by a dense lymphoplasmacytic cuff. Each nodule was composed of myxoid stroma and comprised ovoid or spindle cell fascicles with mild atypia. The mitotic index was very low. Immunohistochemically (Figure 3(a)–(d)), spindle cells were positive for vimentin, CD99, and CD68, and partially positive for desmin. All tumor cells were negative for cytokeratin AE1/AE3, epithelial membrane antigen (EMA), α smooth muscle actin (αSMA), CD34, and S‐100. The Ki‐67 proliferative index was 9.3%. *EWSR1*, *CRBE1*, and *ATF1* gene FISH analysis (Figure 3(e)) showed split signals of 70%, 82%, and 0% respectively. Finally, a diagnosis with AFH with *EWSR1‐CREB1* fusion was made.

## DISCUSSION

AFH is a rare neoplastic disease that usually occurs in the deep dermis or subcutis. Its occurrence in extrasomatic soft tissue sites is rare but has been increasingly reported, such as the brain, lung, mediastinum, retroperitoneum, omentum, ovary, vulva, and bone.^2^ Until now, only one case of primary AFH in the PA has been reported, by Ghigna et al. in 2012, of a 76‐year‐old woman with microscopic features of AFH with *EWSR1‐ATF1* fusion.^3^ The current report is the first case of AFH arising from the ascending artery with *EWSR1‐CREB1* fusion.

Histological features of AFH are distinctive, such as multinodular growth, and up to 80% of cases are surrounded by a dense lymphoplasmacytic infiltrate cuff.^4^ These characteristics are thought to be the best clue to diagnose AFH. Each nodule is composed of a wide morphologic spectrum, and the only constant finding is of sheets and fascicles of ovoid, epithelioid, or spindle cells with bland, vesicular nuclei. AFH lacks a specific immunoprofile, therefore immunohistochemistry is supportive rather than diagnostic. Half of these neoplasms express desmin, which may be diffuse or focal. Occasionally, other markers of myoid differentiation such as SMA, are also expressed. The expression of epithelial membrane antigens CD99 and CD68 has been reported. Vascular endothelial markers such as CD31, CD34, factor‐VIII‐related antigen, S100 protein, and cytokeratin are negative. The Ki‐67 proliferative index is usually low, ranging from 2% to 4%.^2^ *EWSR1* rearrangement is broadly available for subtyping sarcomas. *EWSR1‐CREB‐*family fusion shows a myxoid component, which was previously diagnosed as consistent with extraskeletal myxoid chondrosarcoma (EMC). Recently, EMC has been reported to carry the translocation of the NR4A3 gene,^5^ but other myxoid mesenchymal neoplasms do not belong to the EWSR1‐NR4A3 category. AFH is one of the *EWSR1‐CREB‐*family fusion tumors, especially carrying *EWSR1‐ATF1* or *EWSR1‐CREB1*.^6^

Primary pulmonary myxoid sarcomas (PPMS) also carry *EWSR‐CREB1* fusion. PPMS and AFH sometimes overlap histological and immunohistochemical features. Both can be nodular or multinodular and are composed of spindle or epithelioid cells with bland nuclei and sometimes the same features of immunostaining. However, the following features may help to distinguish PPMS and AFH. First, PPMS has abundant myxoid stroma but AFH is usually focal, <10% of the tumor size. Second, the salient feature of AFH that is not seen in PPMS is peritumoral lymphoid cuff.^7^

Due to the rarity, no standard guidelines have been established for the treatment. According to previous reports, its prognostic factor is the presence of metastatic lesions. If curative surgical treatment can be performed, long‐term prognosis could be expected. Local recurrence occurs in up to 11%^8^ and <5% have been reported to metastasize.^4^ Therefore, WHO proposes AFH as an intermediate biological behavior tumor. AFH sometimes shows pleomorphism and mitotic activity. However, these features are not correlated with clinical behavior^4^ and prognosis, at least during a short follow‐up period.^9^ For the same reason, significant metabolic uptake on PET/CT scan, which usually means high‐grade activity, may not be related to the prognostic measurement. Surgical procedures with clean margins are only related to prolonged overall survival.

In conclusion, we describe a tumor with *EWSR1‐CREB1* fusion occurring in a very rare location.

## DISCLOSURE

The authors report no conflicts of interest in this work.
